# Charge Separation in BaTiO_3_ Nanocrystals: Spontaneous Polarization Versus Point Defect Chemistry

**DOI:** 10.1002/smll.202206805

**Published:** 2023-01-22

**Authors:** Ellie Neige, Thomas Schwab, Maurizio Musso, Thomas Berger, Gilles R. Bourret, Oliver Diwald

**Affiliations:** ^1^ Department of Chemistry and Physics of Materials Paris‐Lodron Universität Salzburg Jakob‐Haringerstrasse 2a Salzburg 5020 Austria

**Keywords:** barium titanate nanoparticles, charge carrier recombination, defect engineering, ferroelectricity, spontaneous polarization

## Abstract

The fate of photogenerated charges within ferroelectric metal oxides is key for photocatalytic applications. The authors study the contributions of i) tetragonal distortion, responsible for spontaneous polarization, and ii) point defects, on charge separation and recombination within BaTiO_3_ (BTO) nanocrystals of cubic and tetragonal structure. Electron paramagnetic resonance (EPR) in combination with O_2_ photoadsorption experiments show that BTO nanocrystals annealed at 600 °C have a charge separation yield enhanced by a factor > 10 compared to TiO_2_ anatase nanocrystals of similar geometries. This demonstrates for the first time the beneficial effect of the BTO perovskite nanocrystal lattice on charge separation. Strikingly, charge separation is considerably hindered within BTO nanoparticles annealed ≥ 600 °C, due to the formation of Ba–O divacancies that act as charge recombination centers. The opposing interplay between tetragonal distortion and annealing‐induced defect formation inside the lattice highlights the importance of defect engineering within perovskite nanoparticles.

## Graphical abstract

Light induced charge separation in BaTiO_3_ nanocrystals: the interplay between tetragonal lattice distortion and annealing induced defect formation highlights the importance of defect engineering within perovskite nanoparticles.

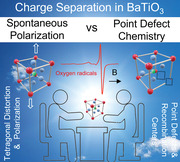

## 1 Introduction

Spontaneous polarization in ferroelectric materials can promote band‐bending and significantly influence surface photochemistry by inhibiting charge recombination, increasing photogenerated charge lifetime, and, hence, the probability for their chemical utilization. To investigate such effects in more detail, BaTiO_3_ (BTO) nanoparticles are used as a reference material.^[^ ^1^, ^2^, ^3^, ^4^, ^5^ ^]^ While the perovskite BTO paraelectric cubic structure does not sustain spontaneous polarization, the non‐centro symmetric tetragonal phase provides the opportunity to study ferroelectric effects on charge separation. Until now, most groups have focused on the materials’ ferroelectric response to explain the changes observed in charge separation and photocatalytic activities of BTO‐based photocatalysts.^[^ ^6^, ^7^, ^8^ ^]^ However, the additional influence of point defects, found within most metal oxide nanoparticles,^[^ ^9^ ^]^ on the BTO photochemical activity is still lacking. In particular, the creation of point defects under specific synthesis conditions that can act as charge recombination centers has been mostly ignored with these materials systems.

Our work intends to fill this gap by studying the influence of ferroelectric effects and point defects on charge separation within various BTO nanoparticles. The photogenerated charge separation yield was quantified by combining O_2_ photoadsorption experiments with electron paramagnetic resonance (EPR), using molecular oxygen as an electron scavenger. Compared to the reference TiO_2_ anatase nanoparticles of similar size and surface area, BTO nanoparticles annealed at 600 °C have a charge separation yield that is > 10 times higher. This is attributed to the beneficial effect of the perovskite lattice on charge separation. Strikingly, we also report a counter‐intuitive decrease in charge separation yield with increasing tetragonal BTO particle size, due to the formation of point defects during the annealing process that acts as recombination centers.^[^ ^10^ ^]^

## 2 Results and Discussion

**Figure 1:**
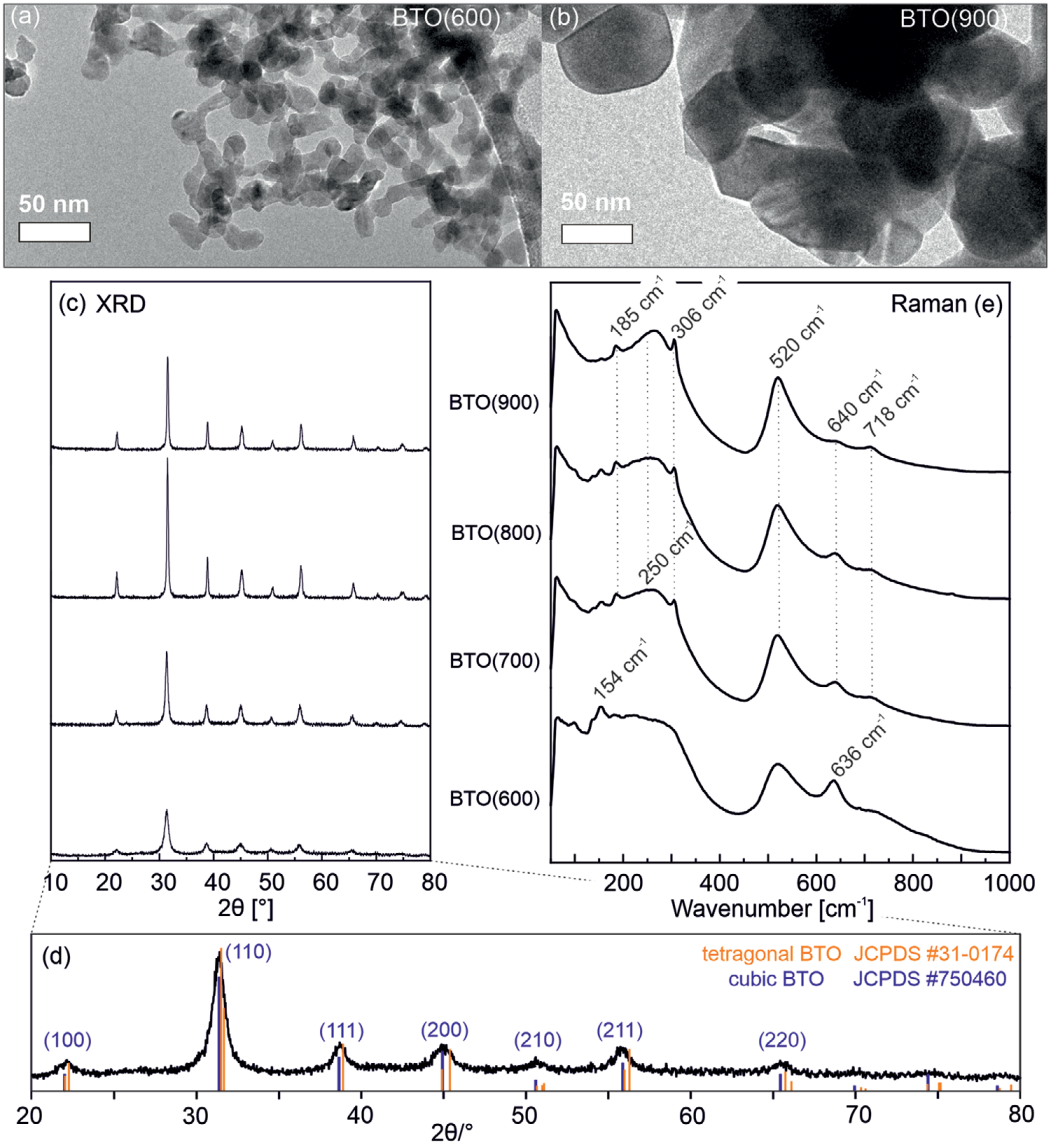
a,b) Representative Transmission electron micrographs, c,d) X‐ray diffraction patterns, and e) Raman spectra of BaTiO_3_ nanoparticle powders that were grown by flame spray pyrolysis (FSP) and subsequently annealed in alternating vacuum/oxygen atmospheres to T = 600 °C, BTO (600), and to temperatures between 700 and 900 °C, BTO (700), BTO (800), and BTO (900). The magnified diffraction pattern at the bottom corresponds to BTO (600).

BaTiO_3_ nanoparticles, synthesized via flame spray pyrolysis (FSP, details in the Experimental Section), were subjected to thermal treatment at temperatures ranging from 600 to 900 °C in alternating cycles of continuous pumping (p < 10^−5^ mbar) and in an oxygen atmosphere (20 mbar) to remove residual carbon, surface hydroxyls and water (Figure S1, Supporting Information). The resulting nanoparticles are equiaxed, non‐faceted, and crystalline (**Figure** **1**a,b). X‐ray diffraction (XRD) patterns (Figure 1c) reveal a cubic crystal structure with broadened diffraction features that depend on the annealing temperature. Annealing at 700, 800, and 900 °C results in a reduction of the diffraction peak widths that indicates crystallite domain size growth.

Annealing at 600 °C gives rise to BTO particles with an average crystallite domain size of ≈11 nm, consistent with the average particle size of 13 nm measured via TEM (Figure S1, Supporting Information). Annealing at higher temperatures leads to larger particles with sizes of 16, 21, and 26 nm for BTO (700), BTO (800), and BTO (900), respectively (Figure S1, Supporting Information). The diffraction features at 2Θ = 75° and 79° and measured on samples after annealing at ≥ 700 °C show a splitting (not shown here),^[^ ^10^ ^]^ which provides clear evidence of the tetragonal crystal phase.^[^ ^11^, ^12^, ^13^ ^]^ The broad and unresolved diffraction features measured on BTO (600) (bottom of Figure 1d), however, point to the prevailing paraelectric cubic phase, but do not allow for unambiguous identification of contributions from the tetragonal phase.

To complement the XRD results, Raman measurements were performed (Figure 1e). All spectra reveal bands at 718 and 520 cm^−1^, which are consistent with those reported for BTO in cubic and/or tetragonal crystal modification.^[^ ^5^, ^14^, ^15^, ^16^ ^]^ Samples annealed at temperatures ≥ 700 °C give rise to spectra with bands at 306 cm^−1^ (sharp) and 250 cm^−1^ (broad, Figure 1e). These are attributed to B_1_, E(TO+LO), and A_1_(TO) modes, respectively, and indicate the presence of non‐centrosymmetric regions that would correspond to tetragonal phase contributions. The bands at 520, 250, and 185 cm^−1^ are assigned to the fundamental TO mode of A_1_ symmetry.^[^ ^17^ ^]^ The spectrum of smaller‐sized particles related to BTO (600) is different and the bands at 185 and 306 cm^−1^ show very low intensities, which suggests that the tetragonal phase represents only a very small fraction of the entire nanocrystal lattice.^[^ ^15^, ^16^ ^]^

**Figure 2:**
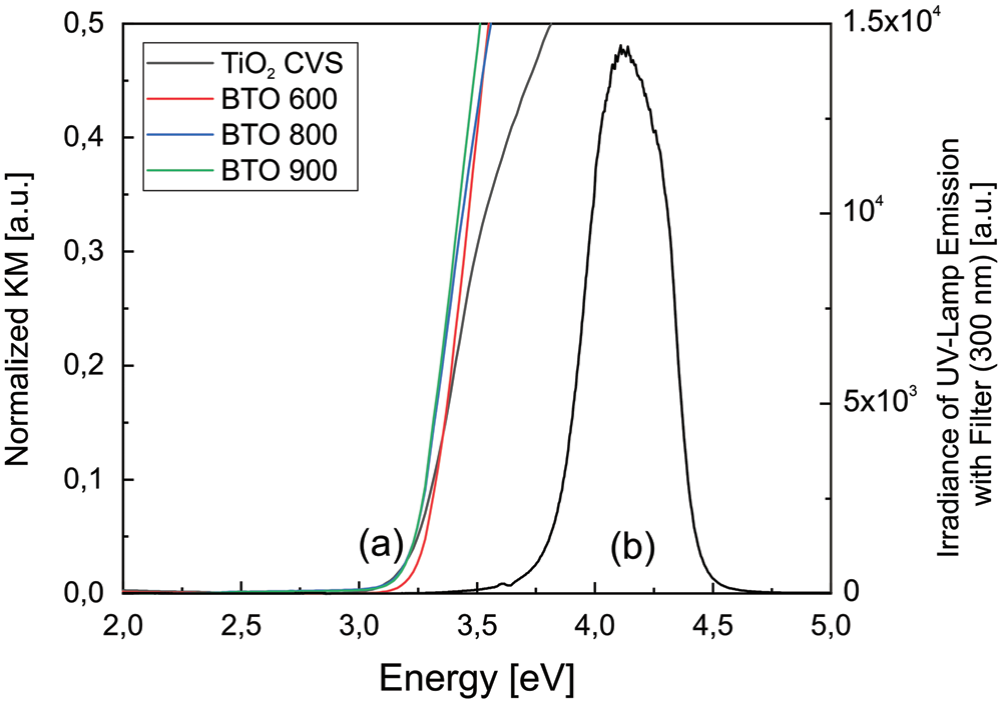
a) UV–vis diffuse reflectance spectra of BaTiO_3_ nanoparticle powders after annealing to temperatures in the range between T = 600 °C (BTO (600)) and 900 °C (BTO (900)) and in comparison to a TiO_2_ anatase (600) nanocrystal powder. (The particle size distribution of TiO_2_ as a reference system coincides with that of BTO (600).) b) emission profile of the UV excitation source used, that is, a high‐pressure xenon lamp equipped with interference and a water filter. With a maximum at hν = 4.13 eV the emission profile has an integral light power density of 950 µW.cm^−2^.

The charge separation yields of the different TiO_2_ and BTO nanoparticle powders were quantified under light irradiation using a high‐pressure Xe lamp equipped with an interference filter centered at 4.13 eV, corresponding to a wavelength of around 300 nm (**Figure** **2**b).

The UV diffuse reflectance (DR) spectra (Figure 2a) of the BTO samples and TiO_2_ anatase nanocrystals are similar, showing an absorption onset at 3.2 eV (or λ = 380 nm) which is clearly below the emission maximum of the UV excitation source (Figure 2b).

The investigation of radiation‐induced paramagnetic defects and oxygen species in the bulk and at the surface of metal oxides with electron paramagnetic resonance (EPR) spectroscopy is a well‐established method.^[^ ^18^ ^]^ During photoexcitation in an O_2_ atmosphere, superoxide anions, that is, O_2_^−^ radical species, form upon interfacial transfer of the photogenerated electrons from the photoexcited particle bulk to gaseous molecular oxygen that acts as an electron scavenger. The *g*‐parameters of the oxygen radicals and, if available, their associated hyperfine data, can be used as a diagnostic value to identify the position and local environment of the oxygen‐centered radicals.^[^ ^19^, ^20^, ^21^, ^22^, ^23^, ^24^ ^]^Moreover, such O_2_^−^ radicals are EPR probes to quantify charge separation ^[^ ^19^, ^20^, ^21^, ^22^, ^23^, ^24^, ^25^ ^]^ and as such are well‐suited to identify any beneficial effect of spontaneous polarisation on the photoinduced reactivity of BTO nanoparticles.

**Figure 3:**
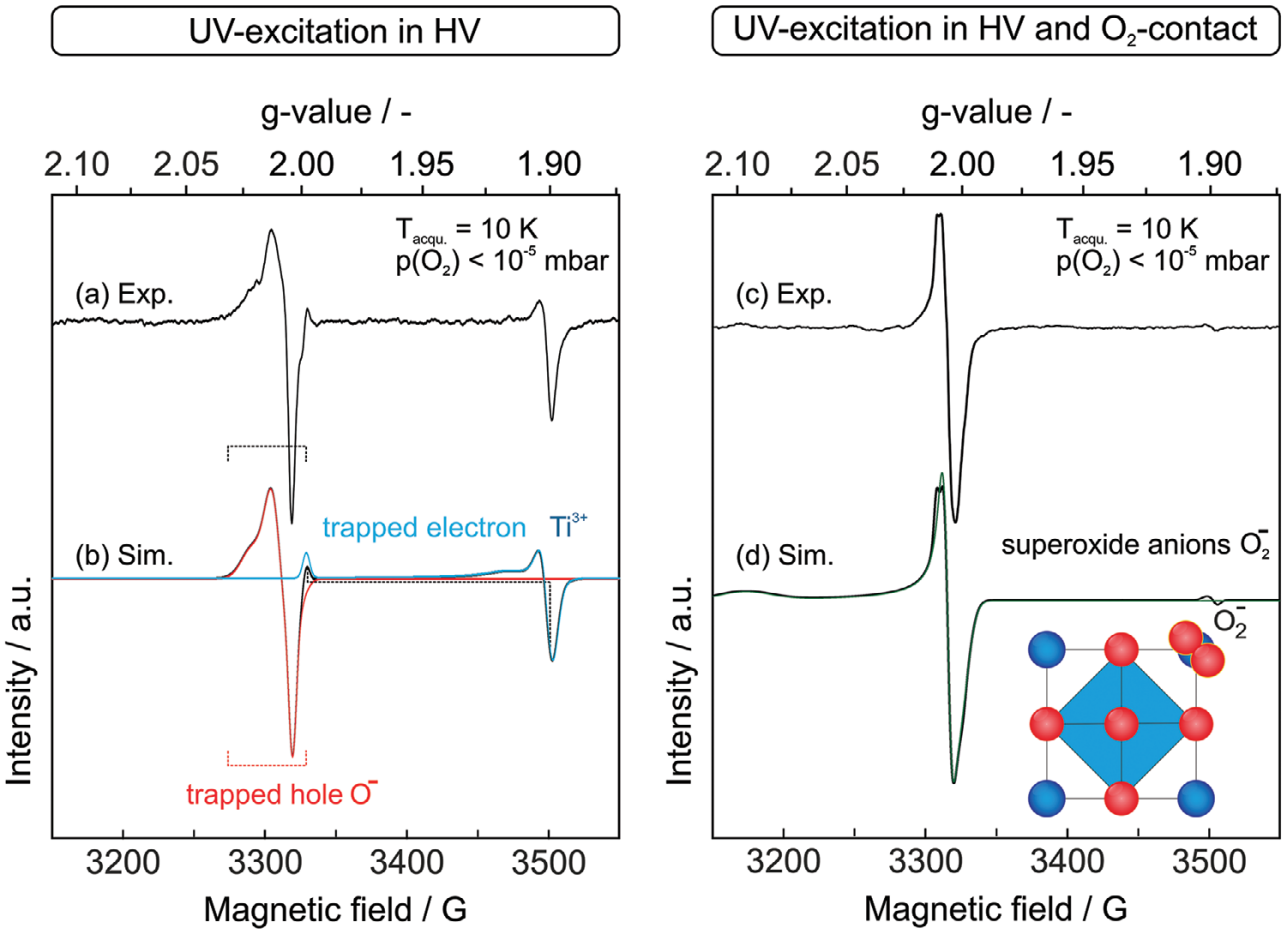
Representative Electron Paramagnetic Resonance (EPR) spectra acquired on BTO (600) nanoparticle powders a) after 60 minutes of UV exposure (hν = 4,13 eV) at 10 K (left panel) in vacuum and c) after subsequent contact with O_2_ gas at room temperature in the dark followed with a further evacuation step to measure again at 10 K and in oxygen‐free atmosphere (right panel). In (b) (left panel) the traces in red and blue and in (d) (right panel) green correspond to the simulated single component spectra.

Prior to UV excitation the EPR spectra of the powdered samples did not show any type of paramagnetic signal. First, we evaluated the impact of UV excitation at 4.13 eV on the paramagnetic sample properties in vacuum. The results, obtained on BTO (600) samples after UV excitation at 10 K, are shown in the left panel of **Figure** **3**. Apart from variations of the relative abundance of different paramagnetic species, that is, Ti^3+^ sites as trapped electrons (blue trace in Figure 3b corresponds to simulated single‐component spectrum) and O^−^ radicals as trapped hole centers (red trace as a simulated single‐component spectrum), the spectrum shown here is representative of the spectra measured on all the BTO nanoparticle powders prepared and analyzed in this study (**Table** **1**).^[^ ^10^ ^]^ The good agreement obtained between our experimental (Figure 3a) and simulated spectra (Figure 3b,d) further confirms our assignments.

**Table 1:** *g* parameters of paramagnetic oxygen species isolated on BaTiO_3_ nanoparticles, obtained from EPR spectrum simulation. (Further details are provided in the Supporting Information).

Spin center	g‐tensor principal values		
	**g**_⊥_	**g**_∥_	
O^−^trapped hole center (axial symmetry)	2.012(6)	2.001(6)	
	**g**_**xx**_	**g**_**yy**_	**g**_**zz**_
Ba^2+^ ∙ O_2_^−^ superoxide anion (orthorhombic symmetry)	2.003(0)	2.008(8)	2.1(0)
	**g**_⊥_	**g**_∥_	
O_2_^−^ superoxide anion (axial symmetry)	2.047(0)	2.026(0)	

Thus, in vacuum and in the absence of electron and hole scavengers (Figure 3b) photoexcitation of BTO nanocrystals yields trapped hole centers *h* ^+^ and electrons *e* ^−^:

(1) $$\begin{array}{c} h\nu +\text{BTO}\;\to \;h^{+}\;\left(O^{2-}\to O^{-}\right)+\;e^{-}\;\left({\text{Ti}}^{4+}\to {\text{Ti}}^{3+}\right) \end{array}$$

Subsequent sample contact of the photoexcited BTO sample with molecular oxygen at room temperature and in the dark, followed by evacuation and cooling to the EPR spectrum acquisition temperature at 10 K, leads to spectral changes that are consistent with the annihilation of *Ti* ^3 +^ centers by molecular oxygen ^[^ ^10^ ^]^ to generate surface adsorbed $O_{2}^{-}$ (Figure 3c,d, right panel). The respective g‐tensor (Table 1) reveals spin centers with orthorhombic symmetry (see the green trace in Figure 3d as a simulated single‐component spectrum). The high value for the *g*_zz_ component (i.e., *g*_zz_ = 2.1(0)) is within the range of values that were previously reported for ${\text{Me}}^{2+}\cdot O_{2}^{-}$ adsorption complexes at metal oxide surfaces,^[^ ^20^, ^22^ ^]^ where Me^2+^ is a divalent metal cation that acts as an adsorption site. This suggests that $O_{2}^{-}$ is ionically bound to ${\text{Ba}}^{2+}$ ions of the BTO surface (scheme in Figure 3d and Figure S3, Supporting Information).

**Figure 4:**
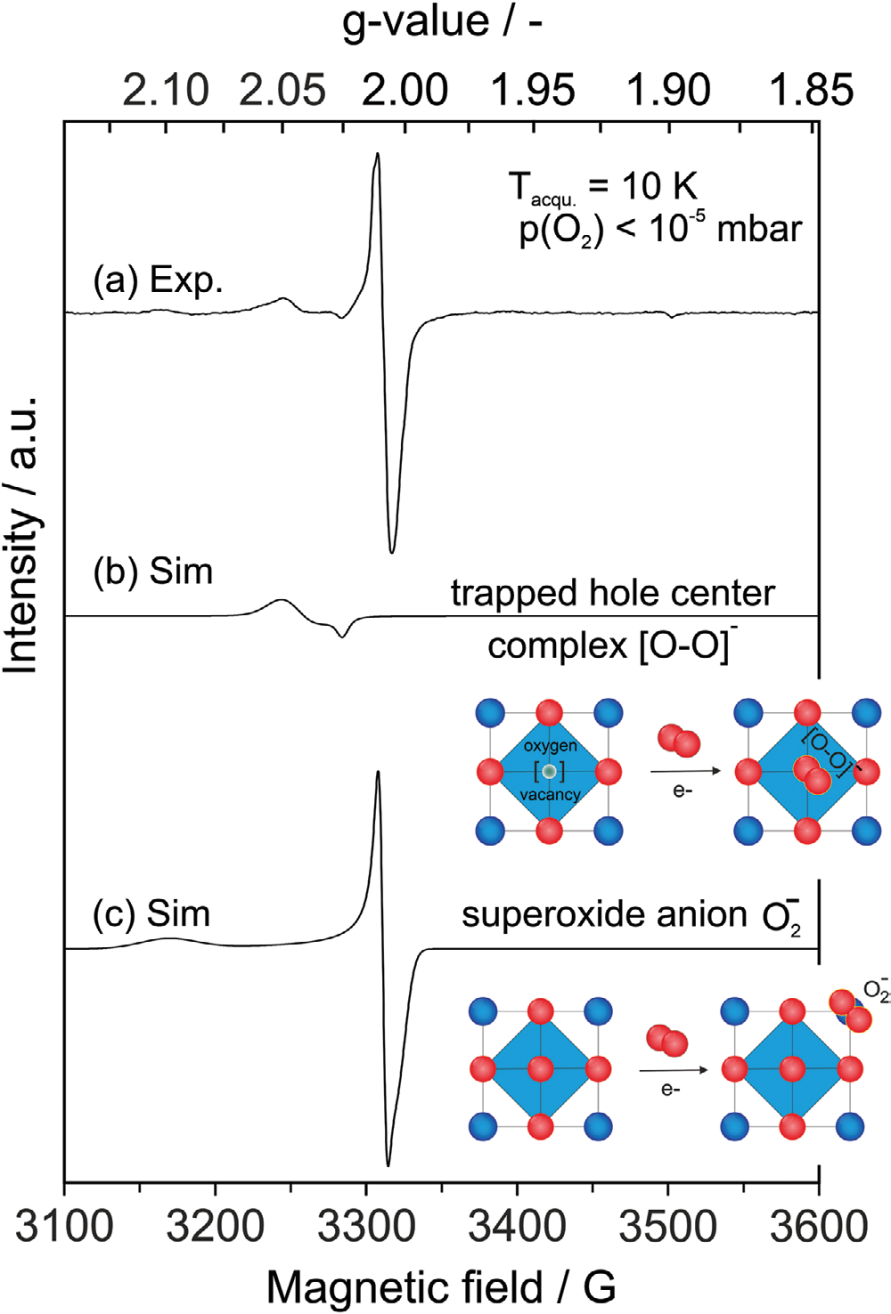
Representative experimental EPR spectrum acquired on BTO (600) after UV excitation in O_2_ atmosphere (a) (P(O_2_) = 30 mbar, *T* = 298 K, *t* = 60 minutes). Traces (b) and (c) are the simulated single component spectra of the trapped hole center complex [*O* − *O*]^−^ and side‐on adsorbed Ba^2+^:O_2_^−^ ions (Figure 3d).

EPR measurement after extended photoexcitation (60 min) of the powder in the O_2_ atmosphere reveals a substantially enhanced concentration of paramagnetic oxygen species along with an altered signal envelope (**Figure** **4**a).

In addition to the fingerprints of the oxygen radicals reported in Figure 3 and Table 1, we measured an axially symmetric broad signal in the low magnetic field range (Figure 4a,b). A similar EPR signature was observed on BTO particle powders that were either electronically reduced by vacuum annealing and subsequently exposed to oxygen gas ^[^ ^10^ ^]^ or measured after photoexcitation in the presence of molecular oxygen. We tentatively attribute this signal to the presence of an end‐on η^1^ $O_{2}^{-}$ adduct stabilized at surface defects of appropriate local potentials, such as oxygen vacancies (scheme in Figure 4b and Figure S4b, Supporting Information with more details on the assignment in the Supporting Information). The (partial) incorporation of one of the oxygen atoms could give rise to a diatomic complex with two electronically and magnetically inequivalent oxygen atoms. However, such a paramagnetic defect should be investigated in more detail, but this goes beyond the scope of this work.

**Figure 5:**
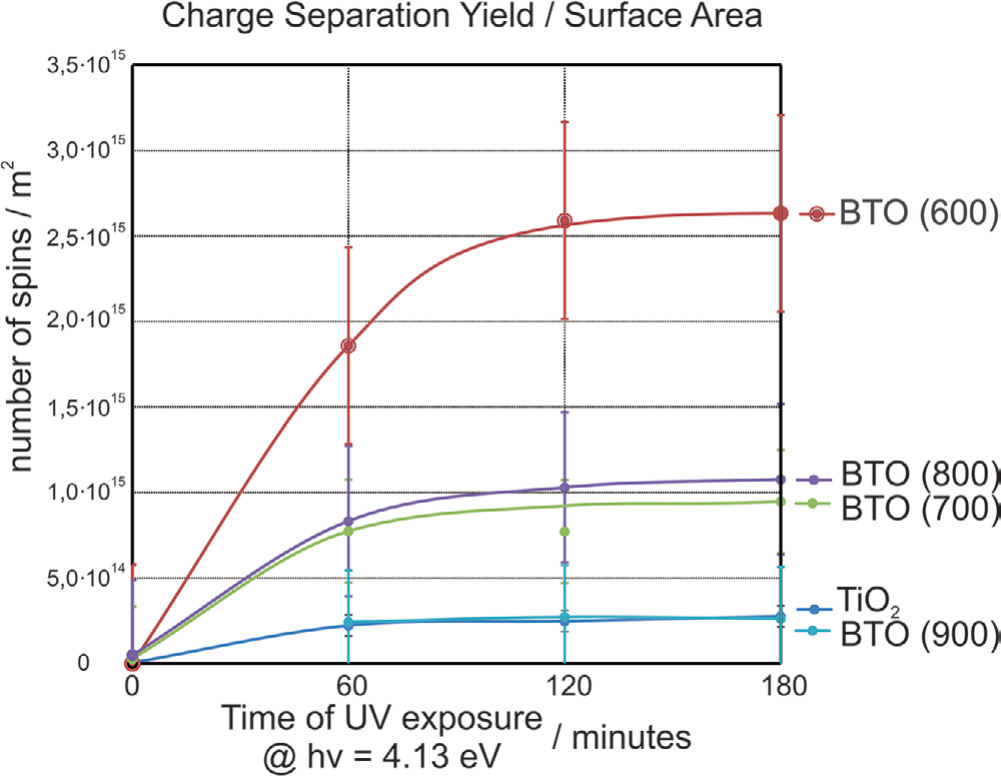
Charge separation yield per surface area determined for TiO_2_ and BaTiO_3_ nanoparticle powders using the detection of oxygen radicals by electron paramagnetic resonance (EPR) spectroscopy (*T*_aqu_ = 10 K) in combination with O_2_ photoadsorption at room temperature. The error bars correspond to the standard deviation of two independently prepared and processed samples. (Alternative plots which show the number of photogenerated charges as a function of sample mass or the estimated number of particles inside the EPR cell are provided in the Supporting Information).

**Figure** **5** plots the yields of photogenerated and trapped charges as determined by EPR spectroscopy on the TiO_2_ anatase and BTO nanocrystal powders under light irradiation and in an O_2_ atmosphere (30 mbar).

For each experiment, the number of spins within the respective particle powder volume was measured and normalized to either the sample surface area (Figure 5), obtained from sorption measurements (Table S1, Supporting Information), the sample mass, or the estimated total number of particles (Figures S5 and S6, Supporting Information). For all samples, the yield of trapped charges increases within the first 1–2 h to different saturation levels. Remarkably, BTO (600) traps 10 times more charges per surface area than TiO_2_ anatase nanoparticles of comparable size distribution and, if similar nanoparticle numbers are compared, comparable total surface area (Figure 5). BTO (700) and BTO (800) show a circa fourfold enhancement in the charge separation yields.

Smaller BTO nanoparticles promote charge separation and enhance photoactivity, as long as they are large enough to be tetragonal and sustain an internal polarization. This trend breaks down as soon as the nanoparticles are small enough to be cubic, leading to reduced photoactivity.^[^ ^26^, ^27^, ^28^ ^]^ Our results do show that the smallest BTO (600) nanoparticles (13 nm in size) have the highest photoactivity. However, BTO (600) is predominantly cubic according to our XRD and Raman measurements (Figure 1c–e). Additionally, the tetragonal BTO (700) and BTO (800) samples, with particle sizes of ≈18 and 30 nm respectively, both show similar and lower charge separation yield than the BTO (600). If the particle size was the main driving force, we would have expected a difference in charge separation between BTO (700) and BTO (800). This is not the case, which suggests that size effects alone are not the main factor affecting charge separation within the nanoparticles synthesized and studied here. Instead, our photoexcitation studies show that BTO (600), which is essentially cubic, can significantly outperform the other tetragonal BTO samples synthesized at higher temperatures because BTO (600) contains the least amount of Ba – oxygen divacancies that act as recombination centers. Thus, our results show that particle size plays a minor role in the charge separation process, while the biggest contribution originates from the presence of different recombination centers.^[^ ^26^, ^27^, ^28^ ^]^

To rationalize the trends in charge separation, one needs to consider the structural and spectroscopic changes the nanoparticle powders undergo during annealing from 600 °C (BTO (600)) to 900 °C (BTO (900)).^[^ ^10^ ^]^ First of all, the BTO (700), BTO (800), and BTO (900) powders show unambiguous XRD and Raman evidence for tetragonal distortion of the lattice (Figure 1c,e), and, thus, of ferroelectric spontaneous polarization, which should enhance charge separation. This could explain the larger charge separation yields of BTO (700) and BTO (800) compared to the paraelectric TiO_2_ nanoparticles. However, the inefficient charge separation yield of the BTO (900) particles that host regions of tetragonal distortion can only be explained by taking point defects into account.

In a previous EPR study, we analyzed the abundance of different paramagnetic point defects on similar BTO nanoparticles.^[^ ^10^ ^]^ Prior to the spectroscopic measurements, the particle powders were subjected to vacuum annealing to make them slightly nonstoichiometric, that is, with deviations in the range 10^−4^ ≤ x ≤ 10^−5^ for BaTiO_3‐_ *_x_*, and to dope the nanoparticles with unpaired electrons as paramagnetic probes. As a key result, we observed that BTO (600) hosts predominantly polaron‐type Ti^3+^ ions surrounded by compressed O^2−^ ion octahedra (blue trace in Figure 3b), while barium–oxygen divacancy complexes, 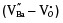,^[^ ^10^ ^]^ prevail in the BTO particles that were annealed at temperatures ≥ 700 °C. These neutral defects are proposed to form above 600 °C, at temperatures at which oxygen vacancies can readily migrate to associate with metal vacancies. It is the annealing‐induced and diffusion‐enabled divacancy formation that explains the variable abundance of different point defect types. The fact that these 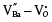 charge‐trapping defects are not observed after photoexcitation (Figure 3a) suggests that they act as recombination centers and annihilate photogenerated holes and electrons at a timescale that cannot be captured by CW X‐band measurements. Comparing the performance of BTO particle powders annealed at 600–800 °C with the TiO_2_ reference, one can clearly see an increase in charge separation yield (Figure 5) due to either a low density of the above‐mentioned point defects on the cubic BTO (600) or possible spontaneous polarization effects that can originate from the more pronounced tetragonal BTO structure of the BTO (700) and BTO (800) samples.

## 3 Conclusion

Herein, we report for the first time the combined effect of spontaneous polarization and point defects on the photochemical activity of BTO nanoparticles. While moderate annealing at temperatures ≤ 600 °C, enhances charge separation yield by a factor > 10 compared to paraelectric TiO_2_ nanoparticles, annealing to higher temperatures (≥ 700 °C) decreases the amount of photogenerated charges. At 900 °C, the beneficial effect of the ferroelectric polarization is completely suppressed, leading to a low yield that is comparable to the one measured on the reference paraelectric anatase TiO_2_ particles.

The outstanding performance of BTO (600) nanocrystals is attributed to the perovskite structure with its intrinsic polarization effects. In the absence of point defects^[^ ^10^ ^]^ that facilitate charge carrier recombination, this gives rise to substantially higher concentrations of trapped charges than for BTO (700), BTO (800), and BTO (900).

This study highlights the need to consider the nature and concentration of point defects in ferroelectric metal oxides to understand and study the resulting photocatalytic properties: The study of ferroelectric properties and spontaneous polarization effects alone is not sufficient. Moreover, our work for BTO nanocrystals shows that the perovskite lattice can provide excellent photochemical performance. These results should be of interest to the many groups working on perovskite catalysts and photocatalysts,^[^ ^6^, ^7^, ^8^, ^29^ ^]^ and should be relevant to current chemical approaches to the synthesis and defect engineering of nanomaterials.^[^ ^30^ ^]^

## 4 Experimental Section

BaTiO_3_ nanoparticles were synthesized by flame spray pyrolysis (FSP) following the work of Schädli et al.^[^ ^4^ ^]^ Barium acetate (1.01704.0500 pro analysis, EMSUREs ACS) was dissolved in 2‐ethylhexanoic acid (EHA) for 3 h at 120 °C and titanium tetraisopropoxide (TTIP, Sigma‐Aldrich, 97%) was diluted in toluene (anhydrous, Sigma‐Aldrich, 99.8%). The two precursor solutions with a Ti:Ba molar ratio of 1:1 and a volumetric ratio between toluene and EHA of 1 were then mixed and injected at a flow rate of 2 mL.min^−1^ by a syringe pump into the nozzle of the flame burner and atomized by the dispersion gas (oxygen). The spray flame in the reactor system is sustained by a supporting flame, which is fuelled by a mixture of methane and oxygen and with oxygen as sheath gas. The obtained nanoparticle powders were then annealed 1 h in vacuum (p < 10^−5^ mbar) at the desired annealed temperature (600, 700, 800, or 900 °C) to remove water and carbon remnants from the samples. An oxygen‐vacuum cycle was then performed, as described in the plots of Figure S1, Supporting Information, to ensure the stoichiometry of the samples.

As titania nanoparticles grown by FSP always contain a small fraction of rutile ^[^ ^31^, ^32^ ^]^ and phase purity is of great importance for the comparison of the charge separation yield with phase‐pure FSP‐grown BaTiO_3_ nanoparticles, the titania nanoparticles were synthesized by metal‐organic chemical vapor synthesis MOCVS.^[^ ^33^, ^34^ ^]^ The titania nanoparticle powders underwent the same annealing treatment as the barium titanate nanoparticle powders annealed at 600 °C (Figure S1a, Supporting Information).

Transmission electron microscopy (TEM) images were obtained using a TVIPS F216 2k by 2k CMOS camera (TVIPS GmbH, Gauting, Germany) on a JEOL JEM‐F200 cold field emission transmission electron microscope (Jeol Ltd, Tokyo, Japan) operating at 200 kV. The samples were measured on copper‐coated lacey carbon grids.

X‐ray diffraction (XRD) data were measured with a Bruker AXS D8 Advance diffractometer using Cu K_α_ radiation (λ = 154 pm). Crystalline domain sizes d_XRD_ were determined from powder diffraction data using the Debye–Scherrer equation.

Raman spectra were recorded using a dispersive Thermo DXR2 Raman microscope (Thermo, USA) equipped with a confocal microscope BX41 (Olympus Corp, Japan) using a 25 µm pinhole entrance slit and a 10x microscope objective. The Raman spectrometer system was operated with the Thermo Omnic acquisition software. The samples were compacted in pellets, and the Raman spectra were obtained with 455 nm laser excitation and a laser power of 3 mW. The nitrogen sorption measurements were performed on an ASAP 2020 adsorption porosimeter (Micromeritics GmbH, Germany). Each sample was degassed under vacuum at 573 K for 3 h prior to the measurements.

Electron paramagnetic resonance (EPR) spectra were acquired using a Bruker EMXplus‐10/12/P/L X‐band spectrometer (Bruker BioSpin, USA) equipped with a waveguide Cryogen‐Free System (Oxford Instruments, United Kingdom). All the spectra were recorded under high vacuum (p < 10^−5^ mbar) and at 10 K with a field modulation frequency of 100 kHz, a modulation amplitude of 0.2 mT, and a microwave frequency of 9.30 GHz. Spin quantification was calculated by the Xenon software from Bruker. For a precise determination of the g factor values related to the paramagnetic defects detected, the spectra were simulated using EasySpin, a MATLAB Toolbox for simulating and fitting EPR spectra.^[^ ^35^ ^]^

The data were not pre‐processed except for normalization. For the size analysis, the number of nanoparticles counted is shown on the cumulative count axis in Figure S2a–d, Supporting Information and was typically larger than 200. For the charge separation yield plot (Figure 5), the error bars of each data point corresponding to the standard deviation of two samples.

## Conflict of Interest

The authors declare no conflict of interest.

## Supporting information

Supporting Information

## Data Availability

The data that support the findings of this study are available from the corresponding author upon reasonable request.
