# Supplementary material for: Charge Separation in BaTiO3 Nanocrystals: Spontaneous Polarization Versus Point Defect Chemistry
Source: Small. 2023 Jan 22;19(16):2206805. doi: 10.1002/smll.202206805 (PMC11475198; doi:10.1002/smll.202206805)
Supplement: Supplementary file 1 — Supporting Information [file SMLL-19-2206805-s001.pdf]

NANO small MICRO

Supporting Information

for *Small,* DOI: 10.1002/smll.202206805

Charge Separation in $BaTiO_3$ Nanocrystals: Spontaneous Polarization Versus Point Defect Chemistry

*Ellie Neige, Thomas Schwab, Maurizio Musso, Thomas Berger, Gilles R. Bourret, and Oliver Diwald**

Supporting Information

Charge Separation in $BaTiO_3$ Nanocrystals: Spontaneous Polarization versus Point Defect Chemistry

Ellie Neige[1], Thomas Schwab[1], Maurizio Musso[1], Thomas Berger[1],

*Gilles R. Bourret[1], Oliver Diwald[1,]**

oliver.diwald@plus.ac.at

[1] Department of Chemistry and Physics of Materials, Paris-Lodron Universität Salzburg, Jakob-Haringerstrasse 2a, 5020 Salzburg, Austria

Particle annealing

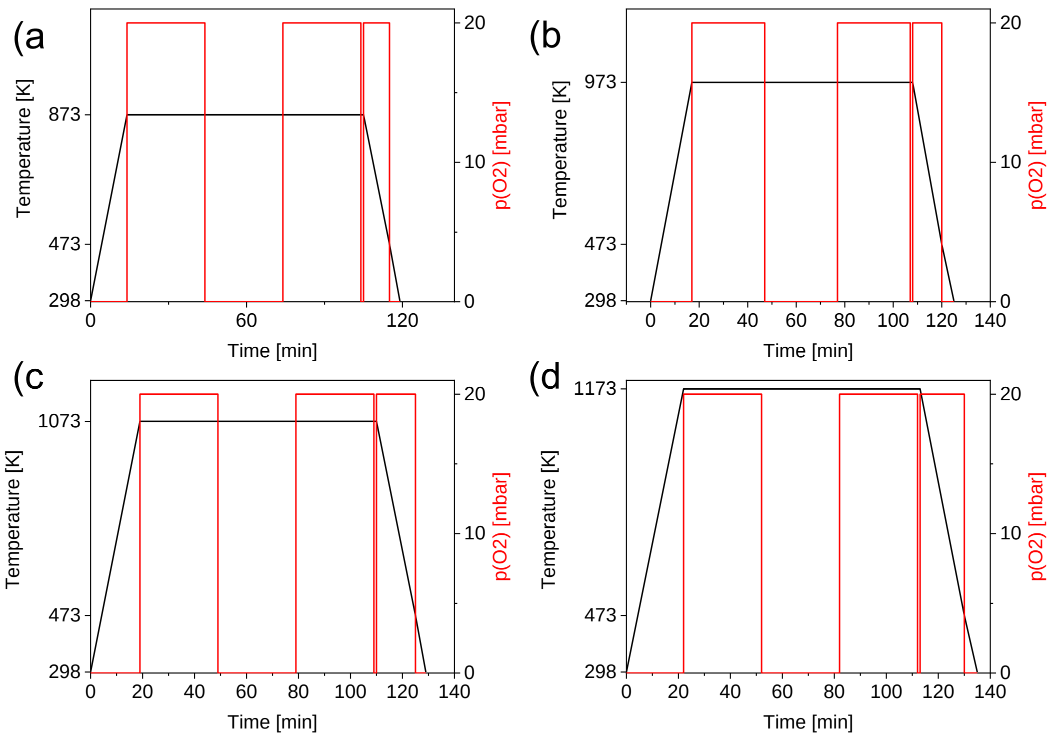

.

Figure S1. Temperature – pressure profiles for the BTO particle powder pre-annealing steps in the temperature range between T = 600 °C **(a)** to T = 900 °C **(d)**.

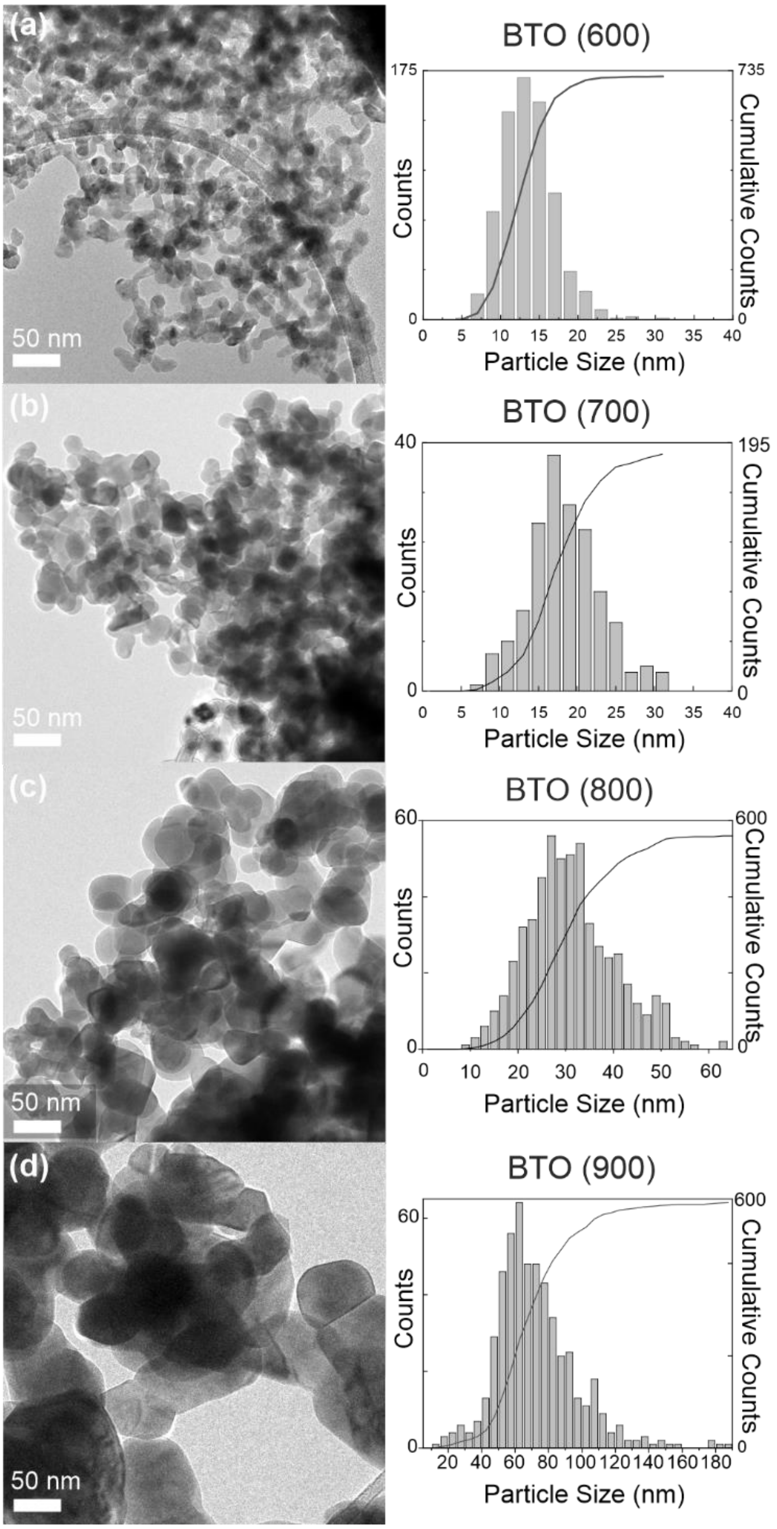

Figure S2. Representative TEM micrographs and particle size distribution (PSD) functions of barium titanate (BTO) nanoparticle powders annealed at different temperatures: **a)** 600 °C (BTO (600)), **b)** 700 °C K (BTO (700)), **c)** 800 °C (BTO (800)) and **d)** 900 °C (BTO (900)).

Materials characterization

Transmission electron microscopy (TEM) images were obtained using a TVIPS F216 2k by 2k CMOS camera (TVIPS GmbH, Gauting, Germany) on a JEOL JEM-F200 cold field emission transmission electron microscope (Jeol Ltd, Tokyo, Japan) operating at 200 kV. The samples were measured on copper-coated lacey carbon grids.

X-ray diffraction (XRD) data were measured with a Bruker AXS D8 Advance diffractometer using Cu K_α radiation (λ = 154 pm). Crystalline domain sizes d_{XRD} were determined from powder diffraction data using the Debye–Scherrer equation.

Raman spectra were recorded using a dispersive Thermo DXR2 Raman microscope (Thermo, USA) equipped with a confocal microscope BX41 (Olympus Corp, Japan) using a 25 µm pinhole entrance slit and a 10x microscope objective. The Raman spectrometer system was operated with the Thermo Omnic acquisition software. The samples were compacted in pellets, and the Raman spectra were obtained with 455 nm laser excitation and a laser power of 3 mW.

The nitrogen sorption measurements were performed on an ASAP 2020 adsorption porosimeter (Micromeritics GmbH, Germany). Each sample was degassed under vacuum at 573 K for 3 hours prior to the measurements.

Electron Paramagnetic Resonance (EPR) spectra were acquired using a Bruker EMXplus-10/12/P/L X-band spectrometer (Bruker BioSpin, USA) equipped with a waveguide Cryogen-Free System (Oxford Instruments, United Kingdom). All the spectra were recorded under high vacuum ($p < 10^{-5}$ mbar) and at 10 K with a field modulation frequency of 100 kHz, a modulation amplitude of 0.2 mT and a microwave frequency of 9.30 GHz. Spin quantification was calculated by the Xenon Software from Bruker. For a precise determination of the g factor values related to the paramagnetic defects detected, the spectra were simulated using EasySpin, a MATLAB Toolbox for simulating and fitting EPR spectra.[2]

Table S1. Surface area values determined by sorption measurements on the BTO nanoparticle powder samples annealed at different temperatures and the chemical vapor synthesized titania samples.

Samples	Specific surface area (m^2/g)
TiO_2 CVS	120
BTO (600)	64
BTO (700)	43
BTO (800)	45
BTO (900)	26

EPR spectroscopy

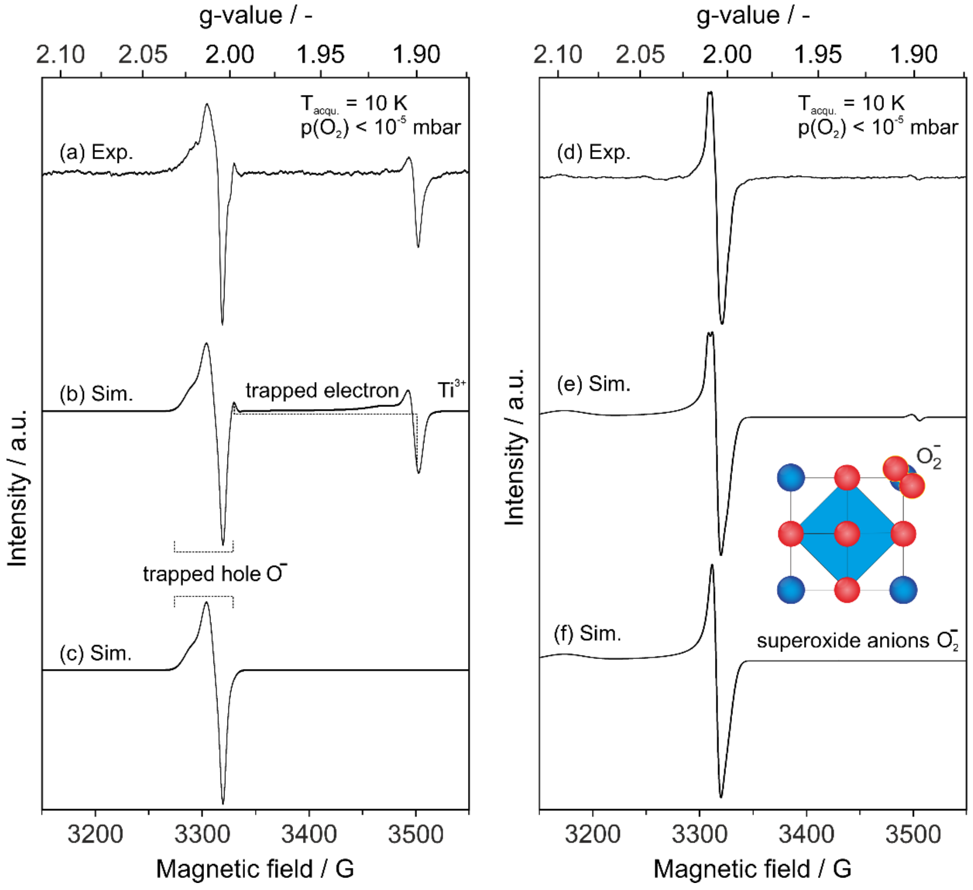

Figure S3: Electron Paramagnetic Resonance (EPR) spectra acquired on BTO (600) nanoparticles after 30 minutes of UV exposure (hν = 4,13 eV) at 10 K (left panel a) and subsequent contact with O_2 gas ($p(O_2)$ = 30 mbar) at room temperature (d), in the dark followed with a further evacuation step for O_2 removal (right panel). Spectra (b) and (e)

correspond the sum of the individual components, which are predominantly trapped hole centers (left panel c) or adsorbed superoxide anions (right panel, f).

Discussion of the end-on η^1 O_2^- adduct (Figure 4 a and c)

The values of the g tensor suggest that the electronic and magnetic structure of the underlying oxygen radical must be different from those reported for electrostatically bound O_2^- species (**Figure 4c**), such as those isolated on the surface of alkaline earth oxides.[3][4][5][6] The high values for the g-tensor components - $g_\perp$= 2.047 and $g_\parallel$ = 2.026 - do not follow the order of values that are typically found for bound superoxol-species with a simple crystal field account, i.e. g_{zz}>> g_{yy}> g_{xx}~ g_e(free spin value).

Similarly large shifts of the g-tensor components, however, were found for O_2^- on different materials systems such as η^2- {Ni·O_2 } · ZSM-5, i.e. a side-on stabilized O_2^- ion bound to Ni(II) ions grafted inside the nanopores of a ZSM-5 zeolite[6], or for O_2^- adducts that can form in the bulk of neutron-irradiated Al_2O_3 single crystals.[7] In the latter case, the principal values of the g-tensor were reported to be g_{zz}= 2.0458, g_{yy} = 2.0096, and g_{zz} = 2.0049. The positive shift of the g tensor components relative to the free spin value, g_e = 2.0023 was attributed to the nature of the underlying paramagnetic defect as a hole center. In this asymmetric diatomic configuration, the complex is composed of one regular and one interstitial oxygen atom and is stabilized by one hole component in the p orbital system. There is currently no similar data acquired on ^{17}O enriched samples, which would provide the hyperfine interaction data necessary for a more detailed analysis of the EPR parameters. As a result, we can only state with confidence that these radicals are related to anionic oxygen species that form at the BTO particle surface upon interfacial electron transfer from the particle to electron accepting oxygen molecules.

However, the presence of an end-on η^1 O_2^- adduct stabilized at surface defects of appropriate local potentials, such as oxygen vacancies (**Figure S3b**), is likely. The (partial) incorporation of one of the oxygen atoms would give rise to a diatomic complex with two

electronically and magnetically inequivalent oxygen atoms. Such a paramagnetic defect should be investigated in more details, but this goes beyond the scope of this work.

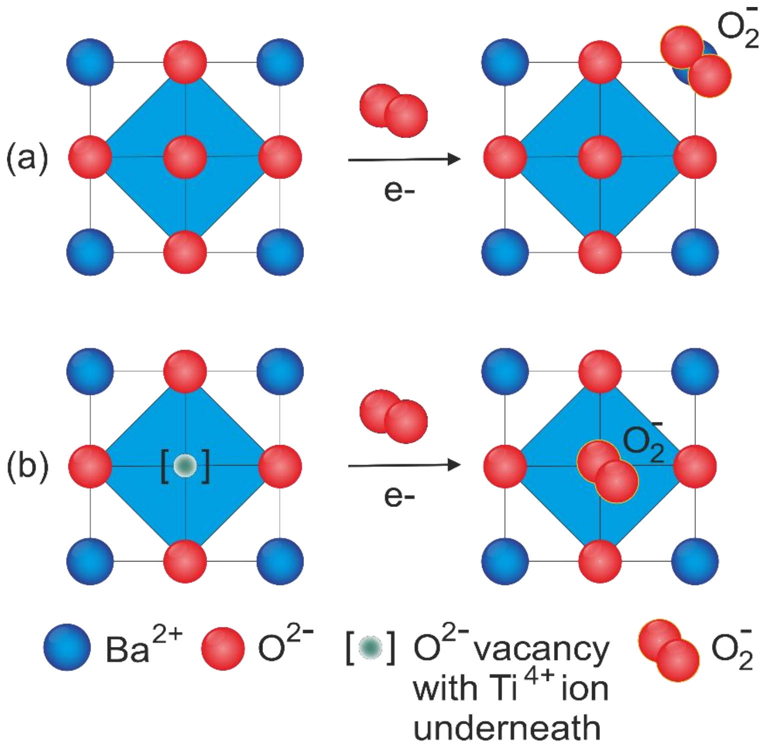

Figure S4: Scheme of a simplified surface element with two potential adsorption sites and adsorbate configurations for O_2^- ions. (a) a side-on η^2 - $Ba^{2+} \cdot O_2^-$ and (b) a hypothesized diatomic oxygen complex which is attached to a surface defect with a local surface potential that can stabilize an end-on η^1 O_2^- configuration.

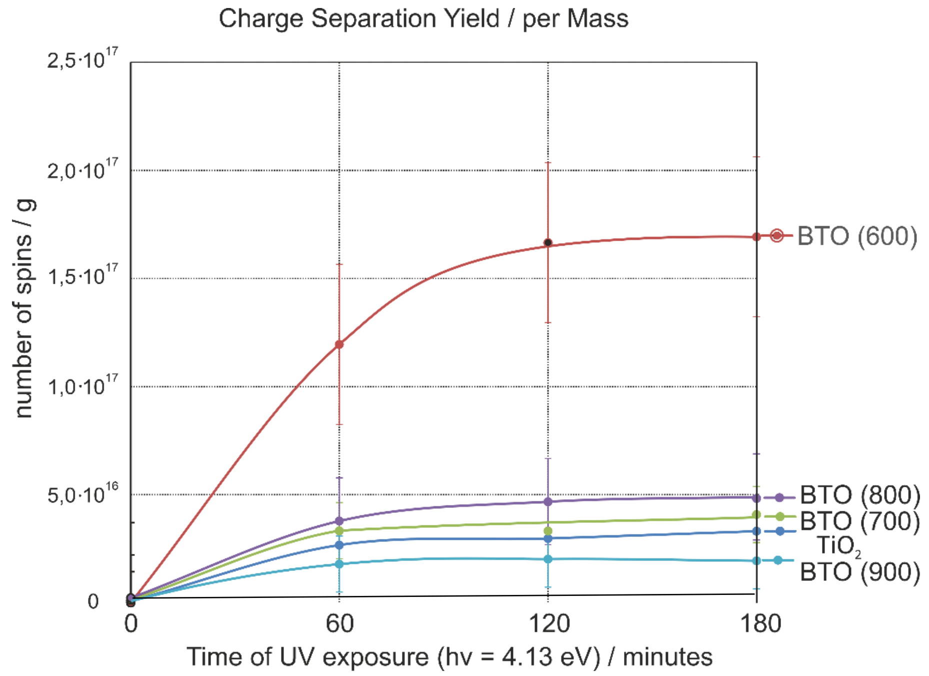

Figure S5: Comparison of the charge separation yield **(number of spins measured in the cavity per mass)** of the CVS titania (dark blue) and the BTO samples annealed to different temperatures (BTO (600) curve in red, BTO (700) in green, BTO (800) in violet and BTO (900) in light blue). The error bars correspond to the standard deviation of two independently prepared and processed samples.

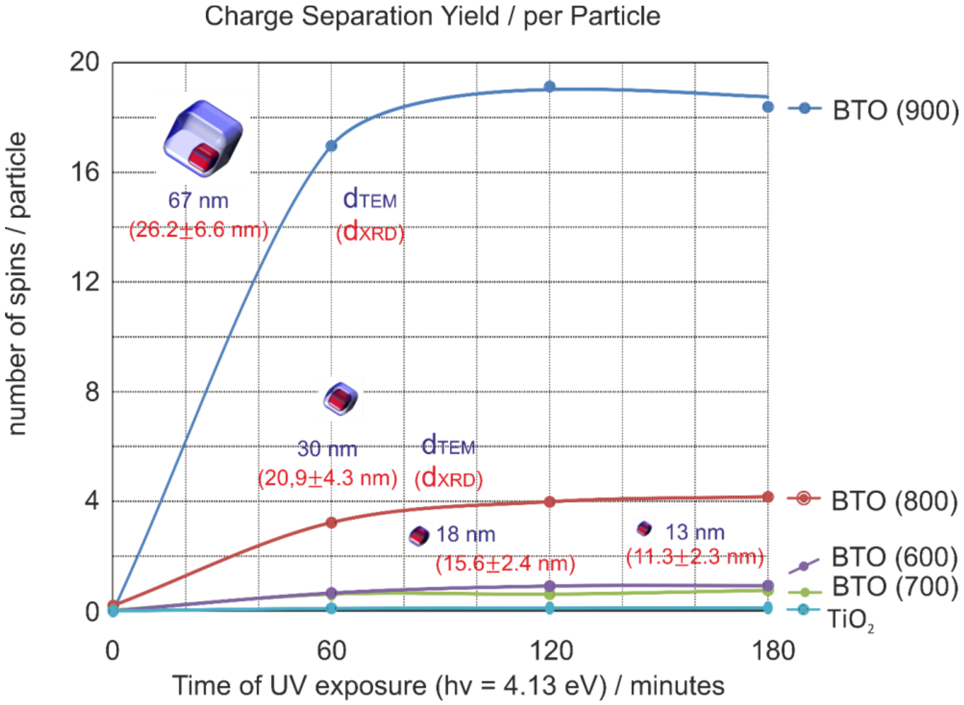

Figure S6: Comparison of the charge separation yield **(number of spins measured in the cavity per particle)** of the CVS titania (light blue) and the BTO samples annealed to different

temperatures (BTO (600) curve in violet, BTO (700) in green, BTO (800) in red and BTO (900) in dark blue).

References

[1] G. N. Schädli, R. Büchel, S. E. Pratsinis, *Nanotechnology* **2017**, *28*, 275705.
[2] S. Stoll, A. Schweiger, *J. Magn. Res. (San Diego, Calif. : 1997)* **2006**, *178*, 42.
[3] M. Chiesa, E. Giamello, *Catal Lett* **2021**, *12*, 31.
[4] M. Anpo, G. Costentin, E. Giamello, H. Lauron-Pernot, Z. Sojka, *J. Catal.* **2021**, *393*, 259.
[5] M. Che, A. J. Tench, *Adv. Catal.***1983**, *32*, 1.
[6] P. Pietrzyk, K. Podolska, T. Mazur, Z. Sojka, *J. Am. Chem. Soc.***2011**, *133*, 19931.
[7] V. Seeman, A. Lushchik, E. Shablonin, G. Prieditis, D. Gryaznov, A. Platonenko, E. A. Kotomin, A. I. Popov, *Sci. Rep.* **2020**, *10*, 15852.
